# c-Kit-Positive Adipose Tissue-Derived Mesenchymal Stem Cells Promote the Growth and Angiogenesis of Breast Cancer

**DOI:** 10.1155/2017/7407168

**Published:** 2017-05-10

**Authors:** Wenjie Li, Haiqian Xu, Cheng Qian

**Affiliations:** ^1^Department of Oncological Surgery, Harbin Medical University Cancer Hospital, Harbin, China; ^2^Plastic and Aesthetic Surgery Center, The First Affiliated Hospital of Harbin Medical University, Harbin, China

## Abstract

**Background:**

Adipose tissue-derived mesenchymal stem cells (ASCs) improve the regenerative ability and retention of fat grafts for breast reconstruction in cancer patients following mastectomy. However, ASCs have also been shown to promote breast cancer cell growth and metastasis. For the safety of ASC application, we aimed to identify specific markers for the subpopulation of ASCs that enhance the growth of breast cancer.

**Methods:**

ASCs and bone marrow-derived vascular endothelial progenitor cells (EPCs) were isolated from Balb/c mice. c-Kit-positive (c-Kit^+^) or c-Kit-negative (c-Kit^−^) ASCs were cocultured with 4T1 breast cancer cells. Orthotropic murine models of 4T1, EPCs + 4T1, and c-Kit^+/-^ASCs + 4T1/EPCs were established in Balb/c mice.

**Results:**

In coculture, c-Kit^+^ ASCs enhanced the viability and proliferation of 4T1 cells and stimulated c-Kit expression and interleukin-3 (IL-3) release. In mouse models, c-Kit^+^ASCs + 4T1/EPCs coinjection increased the tumor volume and vessel formation. Moreover, IL-3, stromal cell-derived factor-1, and vascular endothelial growth factor A in the c-Kit^+^ASCs + 4T1/EPCs coinjection group were higher than those in the 4T1, EPCs + 4T1, and c-Kit^−^ASCs + 4T1/EPCs groups.

**Conclusions:**

c-Kit^+^ ASCs may promote breast cancer growth and angiogenesis by a synergistic effect of c-Kit and IL-3. Our findings suggest that c-Kit^+^ subpopulations of ASCs should be eliminated in fat grafts for breast reconstruction of cancer patients following mastectomy.

## 1. Introduction

Adipose tissue-derived mesenchymal stem cells (ASCs) with autologous fat improve the regenerative ability and retention of fat grafts and are increasingly being used for breast reconstruction of breast cancer patients following mastectomy [1]. However, increasing evidence has shown that ASCs may promote the growth and metastasis of breast cancer cells [2–5], and several studies have demonstrated that ASCs inhibit the growth of breast cancer [6, 7]. These contradictory observations may be due to different sources of ASCs, tumor models, and biomarkers for identifying ASCs. To enhance the safety of ASC application in breast reconstruction, it is very important to identify specific biomarkers to distinguish the breast cancer cell growth-promoting ASC subpopulation from other ASC subpopulations that do not enhance the growth and metastasis of breast cancer cells.

c-Kit is a protooncogene located at chromosome 4q12, and its encoding protein is a transmembrane receptor tyrosine kinase [8, 9]. c-Kit is expressed in many cells of the tumor microenvironment, including mesenchymal, mast, and progenitor cells. In breast cancer, the c-Kit/Kit ligand (KitL) signaling pathway promotes the proliferation, survival, and metastasis of tumor cells [10]. Moreover, the expression level of c-Kit is closely related to triple-negative breast cancer [11]. Recently, it was found that c-Kit^+^ ASCs display a higher differentiation potential in comparison to c-Kit^−^ ASCs [12, 13]. These facts suggest that c-Kit may be a potential biomarker that could distinguish the breast cancer cell growth-promoting ASC subpopulation from other ASC subpopulations.

The growth and metastasis of tumor cells is dependent on vessel formation in the tumor mass [14]. It has been shown that tumor cells recruit bone marrow-derived vascular endothelial progenitor cells (BM-EPCs) by increasing the expression of hypoxia-inducible factor-1*α* (HIF-1*α*) and vascular endothelial growth factor (VEGF), both of which play an important role in angiogenesis [15, 16]. The interaction of EPCs and tumor cells can enhance angiogenesis, which plays a crucial role in the growth and metastasis of tumor cells [17, 18]. ASCs have been demonstrated to promote angiogenesis by secreting growth factors within a variety of tumor types [19–22]. However, since the tumor microenvironment is very complex, whether ASCs differentiate into endothelial-like cells or recruit endothelial cells for vessel formation during tumor angiogenesis remains to be determined.

To explore the role and mechanism of c-Kit^+^ ASCs in breast cancer progression, in this study, we established a coculture model of ASCs and breast cancer cells. Furthermore, we analyzed the impact of c-Kit^+^ ASCs on tumor angiogenesis using breast cancer mouse models.

## 2. Materials and Methods

### 2.1. Cell Culture

4T1 breast cancer cells were purchased from the American Type Culture Collection (Manassas, VA, USA) and cultured in RIPM-1640 medium (Lonza, Walkersville, MD, USA) supplemented with 10% fetal bovine serum (FBS) (Gibco, Grand Island, NY, USA), 2 mM glutamine, 100 U/mL penicillin, and 100 *µ*g/mL streptomycin.

### 2.2. Animals

Four-week-old female nude mice (Balb/c) were obtained from the SLAC Laboratory Animal Corporation (Shanghai, China) and were housed in a specific pathogen-free room. The Animal Committee of Harbin Medical University approved all the experimental protocols and animal handling procedures. All experimental procedures and postoperative animal care were conducted in accordance with the National Institute of Health's Guidelines for the Care and Use of Laboratory Animals.

### 2.3. Preparation of c-Kit^+^ ASCs

Six-week-old Balb/c female mice were sacrificed by cervical dislocation, and inguinal fat tissues were dissected. After mincing the tissues into 2-3 mm pieces, they were digested with 200 U/mL collagenase II (Sigma, St. Louis, MO, USA) for 30 min, followed by centrifugation at 1200 rpm for 10 min. The pellets were sequentially filtered through 200 mesh filters and centrifuged at 12,000 rpm for 10 min. The pelleted cells were washed twice with phosphate-buffered saline (PBS) and resuspended in Dulbecco's modified Eagle medium (DMEM; Lonza) containing 10% FBS. The cells were grown at 37°C in a humidified atmosphere containing 5% CO_2_, and the medium was changed daily for 2-3 days. After 3 weeks of culture, c-Kit^+^/CD90^+^ cells were isolated by magnetic bead separation and further purified by fluorescence-activated cell sorting. The adipogenic differentiation potential of ASCs was routinely induced for 2 weeks using medium supplementation (1 : 1 DMEM/Hams F-12 containing 3% fetal calf serum, 100 nM insulin, 1 *μ*M dexamethasone, and 0.25 mM 3-isobutyl-1-methylxanthine) and determined using oil red O staining, following standard protocols.

### 2.4. Preparation of BM-EPCs

Bone marrow cells were isolated from the femurs of Balb/c female mice and diluted in Histopaque-1083 (Sigma) (7 : 4), immediately followed by centrifugation at 2400 rpm for 25 min at room temperature. The layer of bone marrow cells at the opaque interface was transferred to a tube containing PBS and centrifuged at 1500 rpm for 10 min at 4°C. EPCs from the mononuclear cells were isolated with CD34 and VEGFR2 magnetic bead separation (Miltenyi Biotech Inc., Auburn, CA, USA) and cultured in endothelial cell growth medium-2 (EGM-2; Lonza) at 37°C and 5% CO_2_ in a humidified incubator. Medium was changed daily for 2-3 days.

### 2.5. Immunofluorescence

The adherent cells were trypsinized and plated on EZ slides (Millipore, Billerica, MA, USA) for immunofluorescence assays. The cells were labeled with monoclonal rat anti-CD90 antibody (Abcam, Cambridge, MA, USA), followed by donkey anti-rat secondary antibody conjugated with Alexa Fluor 488 (Thermo Scientific, Waltham, MA, USA). For c-Kit staining, cells were incubated with polyclonal rabbit anti-c-Kit (Abcam) antibody, followed by incubation with goat anti-rabbit secondary antibody conjugated with Alexa Fluor 647 (Abcam). After incubation with 4′,6-diamidino-2-phenylindole (Thermo Scientific) for 1 min, the cells were observed under a fluorescence microscope (Olympus, Tokyo, Japan). The percentages of c-Kit^+^ and CD90^+^ cells during each isolation were analyzed using Image J software.

### 2.6. Direct Coculture of ASCs with 4T1 Cells

c-Kit^+^ or c-Kit^−^ ASCs were cocultured with 4T1 cells at a ratio of 1 : 1. The cells were plated on gelatin-coated (1% in PBS) 24-well plates and cultured in DMEM at a density of 50,000/cm^2^. As controls, 4T1 cells or ASCs alone were cultured under the same conditions. The cells were incubated for 1–5 days at 37°C with humidified 5% CO_2_.

### 2.7. Indirect Coculture of ASCs with 4T1 Cells

In a 24-well Transwell culture plate (Corning, NY, USA), 3 × 10^4^ 4T1 cells were plated in the bottom chamber and 3 × 10^4^ ASCs were plated in the upper chamber in DMEM. The chambers were incubated for 1–5 days in a 37°C incubator with humidified 5% CO_2_.

### 2.8. Tube Formation Assay

Matrigel (BD, Franklin Lakes, NJ, USA) was added to a 96-well plate, 50 *μ*L per well. After incubation at 37°C with 5% CO_2_ for 1 h, ASCs or EPCs (10^4^) in 100 *μ*L of culture medium were added. The cells were incubated for 18 h, and the area of tube formation was recorded using imaging software (Olympus, Tokyo, Japan).

### 2.9. Cell Viability Assay

Approximately 3 × 10^3^ 4T1 cells were cultured in culture supernatant from ASCs in 96-well plates. After 1–5 days, 10 *μ*L of cell counting kit-8 (CCK-8) solution was added per well, and the cells were further incubated at 37°C with 5% CO_2_ for 3 h. Then, the absorbance at 450 nm was recorded using a microplate reader.

### 2.10. Cell Proliferation

DNA quantification was performed to assess cell proliferation using the Quant-iT PicoGreen dsDNA reagent and kit (Invitrogen, Carlsbad, CA, USA). After culturing for 1–5 days, the 4T1 cells in the direct coculture model were washed two times with PBS, and 200 *μ*L of 0.2% (v/v) Triton X-100/5 mM MgCl_2_ was added to the cells. Following digestion, the samples were centrifuged at 12,000 rpm for 10 min at 4°C. The supernatant (100 *μ*L) was transferred to a 96-well plate, and 100 *μ*L of PicoGreen fluorescence reagent (1 : 200 in Tris-EDTA buffer) was added. Fluorescence was measured in a microplate reader (Nanodrop 3300, Thermo Scientific), and values were calculated using known DNA standards.

### 2.11. RNA Extraction and Quantitative Real-Time Polymerase Chain Reaction (qPCR)

Total RNA was extracted using RNeasy Mini Kits (QIAGEN, Düsseldorf, Germany), according to the manufacturer's protocol. Total RNA (1 *μ*g) was reverse-transcribed into cDNA using a QuantiTect Reverse Transcription Kit (QIAGEN). The primer sequences used were as follows: mouse c-Kit forward, 5′-CTGACGTGCATTGATCCCGA-3′, reverse, 5′-CTCGTGAGGCCATTGCTGTT-3′; GAPDH forward, 5′-AGGTCGGTGTGAACGGATTTG-3′, reverse, 5′-GGGGTCGTTGATGGCAACA-3′. The qPCR was performed on a CFX900 thermal cycler (Bio-Rad, Hercules, CA, USA) using THUNDERBIRD SYBR PCR Mix (TOYOBO, Osaka, Japan), according to the manufacturer's protocol. GAPDH was used as an internal control. Amplification parameters were as follows: one cycle of 50°C for 2 min, 95°C for 3 min, followed by 40 cycles of 95°C for 10 s and 60°C for 30 s. Relative expression was determined using the comparative threshold cycle method (2^−ΔΔCt^).

### 2.12. Western Blot Analysis

Total proteins were extracted using a total protein extraction kit (Vazyme Biotech, Nanjing, China), and concentration was measured using a bicinchoninic acid protein assay kit (Beyotime Biotechnology, Shanghai, China). A total of 20 *μ*g of protein was separated by 7.5% sodium dodecyl sulfate-polyacrylamide gel electrophoresis and transferred onto a polyvinylidene difluoride membrane. The membrane was incubated in blocking buffer containing 5% nonfat dry milk in Tris-buffered saline and Tween 20 (10 mM Tris-HCl, pH 8.0, 100 mM NaCl, and 0.05% Tween) for 1 h at room temperature. After incubating the membrane with primary antibody (rabbit anti-c-Kit antibody diluted at 1 : 3000) overnight at 4°C and horseradish peroxidase- (HRP-) conjugated goat anti-rabbit antibody (1 : 4000, Abcam) for 2 h at room temperature, protein-antibody complexes were visualized with an Enhanced Chemiluminescence Western Blotting Detection Kit (Beyotime Biotechnology, Shanghai, China) and analysis system (Bio-Rad). *β*-Actin was detected by the same method as a loading control.

### 2.13. Enzyme-Linked Immunosorbent Assay (ELISA)

Cell culture supernatant was collected after culturing for 1, 3, and 5 days by centrifugation for 20 min at 1000 rpm. Primary tumor tissues were rinsed in ice-cold PBS to remove excess blood and weighed. The tissues were minced into small pieces and crushed with liquid nitrogen in a mortar. The resulting suspension was sonicated and centrifuged for 5 min at 5000 rpm. The supernatant was immediately subjected to cytokine and chemokine detection with a commercially available ELISA kit (Cloud-Clone Corp., Houston, TX, USA), according to the manufacturer's protocols.

### 2.14. Animal Model

Subcutaneous orthotopic injection in 4-week-old female nude mice (BALB/c) was performed under general anesthesia (1.2% Avertin, 0.1 mL/10 g). The cells, including 10^5^ 4T1 cells, 10^5^ ASCs, and 10^4^ EPCs, were resuspended in 200 *µ*L of PBS/Matrigel and injected into the mammary fat pads of female nude mice alone or EPCs + 4T1 or ASCs + 4T1/EPCs coinjections. In every group (*n* = 5), the tumor size was measured twice/week, and the tumor volume was calculated according to the following formula: tumor volume = 0.5 × (*D*_max_ × *D*_min_^2^). Three weeks after injection, the mice were sacrificed and the primary tumors were removed for further evaluation.

### 2.15. Immunohistochemistry

After tumor removal, the fresh tumors were immediately embedded in optimum cutting temperature compound (Sakura, Zoeterwoude, Netherlands) and sectioned (5 mm), followed by staining with hematoxylin and eosin (H&E), CD31 antibody, and oil red O. Briefly, sections mounted on slides were dehydrated in ethanol, rinsed in PBS containing Tween 20 (PBST), and incubated with 0.3% hydrogen peroxide for 15 min. After washing with PBST, sections were blocked by incubation in 3% bovine serum albumin for 30 min, followed by overnight incubation with primary antibody (rabbit anti-CD31 diluted 1 : 50; Abcam). Slides were washed with PBST followed by a 1 h incubation with HRP-conjugated goat anti-rabbit secondary antibody (1 : 4000, Abcam), rinsed in PBST, and exposed to 3,3′-diaminobenzidine (Solarbio, Beijing, China). Then, counterstaining was performed with hematoxylin (Solarbio, Beijing, China). For H&E staining, sections were stained in hematoxylin for 3 min, washed in water, and then exposed for 5 min to eosin (Solarbio). The immunostaining results were analyzed using imaging software (Olympus, Tokyo, Japan).

### 2.16. Statistical Analysis

All experiments were performed in triplicate. All data were statistically analyzed using SPSS version 13.0, and graphs were made using GraphPad Prism version 5.0 software. One-way analysis of variance and Newman-Keuls post hoc tests were used to compare variance between groups. A *p* value ≤ 0.05 was considered as a significant difference. Differences were considered highly significant when *p* ≤ 0.01.

## 3. Results

### 3.1. Isolation and Characterization of ASCs and EPCs from Mice

**Figure 1:**
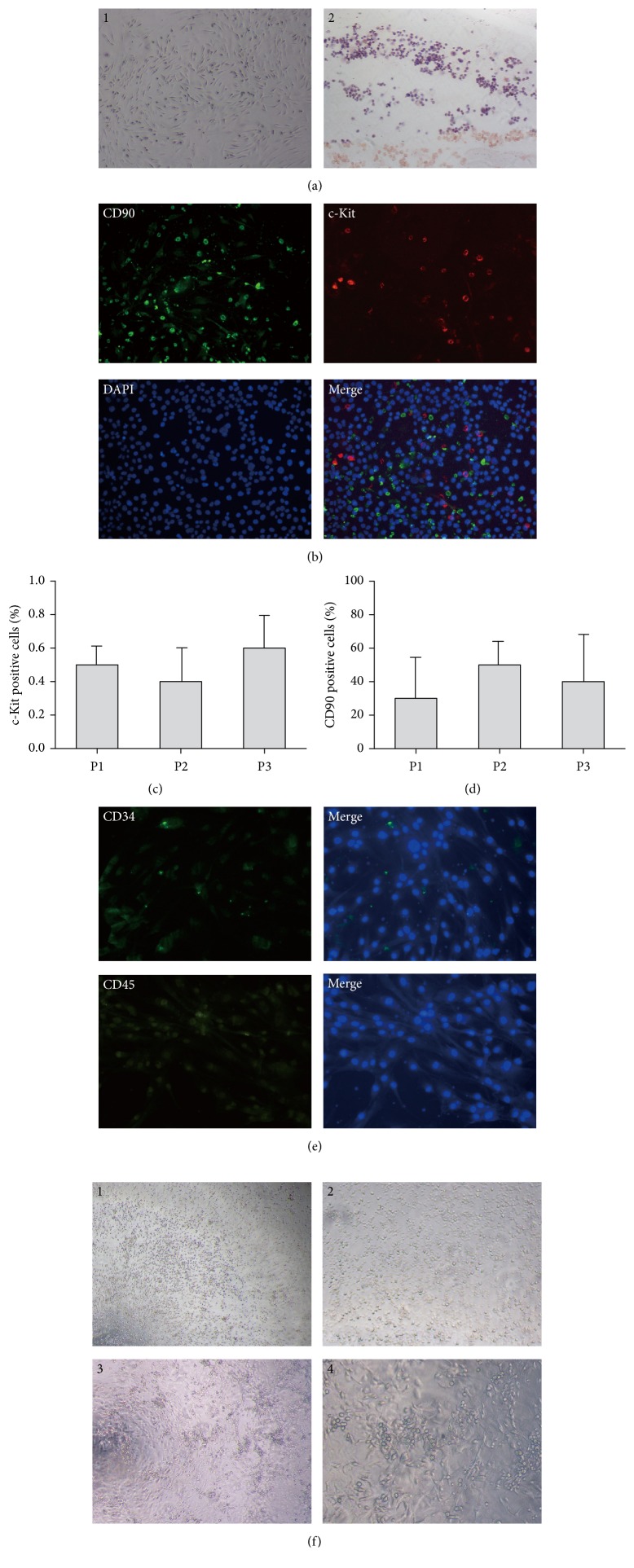
The characterization of isolated ASCs and EPCs. ASCs were isolated from inguinal adipose tissue of Balb/c female mice and cultured in DMEM. The cells were placed on EZ slides for detection of biomarker expression using immunofluorescence. BM-EPCs were isolated from the femurs of Balb/c female mice and cultured in EGM-2. A total of 1 × 10^3^ EPCs were plated on methylcellulose containing EGM-2 medium for 8–10 days, and colony formation units were defined as cluster-like collections of cells. (a) The morphology and differentiation potential of ASCs. ASCs appeared as a spindle shape, and adipogenic differentiation was confirmed by oil red O staining. (b) Isolated ASCs were stained for c-Kit and CD90 expression. ((c)-(d)) The percentages of c-Kit^+^ and CD90^+^ cells during each isolation. (e) Isolated ASCs were stained for CD34 and CD45 expression. (f) The morphology and colony formation assay in EPCs. EPCs cultured for 7–14 days demonstrated a cobblestone appearance on collagen-coated plates and cell-cluster formation on methylcellulose. (a)1, (a)2, (f)1, and (f)3: 40× magnification; (b) and (f)2: 100× magnification; (e) and (f)4: 200× magnification. ASCs: adipose-derived mesenchymal stem cells; EPCs: endothelial progenitor cells.

To investigate whether c-Kit^+^ ASCs promote the growth of breast cancer cells, we isolated ASCs from mouse inguinal adipose tissues. The isolated ASCs appeared as a spindle shape, and oil red O staining showed that adipogenic differentiation of sorted ASCs contained lipid drops inside their cytoplasms, a feature of mature adipocytes (Figure 1(a)). The cells obtained from mouse adipose tissues were mostly CD90^+^ cells and included a c-Kit^+^ subpopulation (Figures 1(b), 1(c), and 1(d)). Nevertheless, there were very few cells that were positive for the endothelial progenitor cell marker CD34, and no CD45^+^ subpopulation was found by immunofluorescence staining (Figure 1(e)). These results indicated that the isolated and expanded cells including c-Kit^+^/CD90^+^ ASCs were not contaminated with endothelial or hematopoietic cells.

To assess whether the interaction of ASCs and EPCs promotes breast cancer angiogenesis, we isolated BM-EPCs from mice. The isolated mononuclear cells appeared as round-shaped cells that attached on plates at days 3–5. At days 7–14, the cells demonstrated a cobblestone appearance on gelatin-coated plates, which is a characteristic of EPCs. In methylcellulose media, EPCs demonstrated cell-cluster formation consistent with the ability to form colonies (Figure 1(f)). These results indicated a successful isolation of EPCs from mouse bone marrow.

### 3.2. c-Kit^+^ ASCs Promote the Viability and Proliferation of Breast Cancer Cells

**Figure 2:**
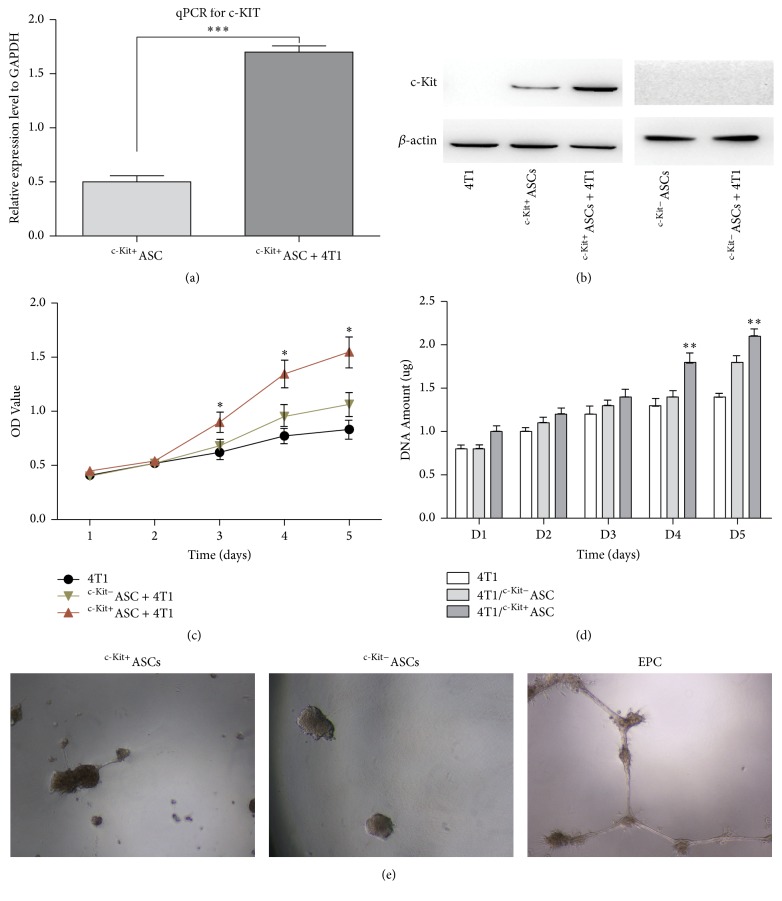
Effects of c-Kit expression of ASCs on 4T1 breast cancer cells. ASCs and 4T1 cells were cocultured in a ratio of 1 : 1. After culturing, RNA and protein were extracted to detect the c-Kit mRNA and protein expression in different culture models. After 1–5 days, the viability and proliferation of 4T1 cells were detected using a CCK-8 assay and DNA quantification, respectively, in indirect coculture experiments. For the tube formation assay, 10^4^ ASCs and 10^4^ EPCs were plated on Matrigel, respectively, and incubated for 18 h, and then the area of tube formation was observed. (a) The mRNA expression of c-Kit was higher in the c-Kit^+^ASCs + 4T1 direct coculture group than in the single culture groups using qPCR, and the expression of c-Kit mRNA was not detected in the 4T1 cells. The qPCR results were normalized against GAPDH. (b) The level of c-Kit protein expression in the c-Kit^+^ASCs + 4T1 direct coculture group was higher than that in the single culture groups by western blot analysis. But no c-Kit protein expression was detected in the c-Kit^−^ ASCs + 4T1 group. Anti-*β*-actin antibody served as a control. ((c)-(d)) The viability and proliferation of 4T1 cells were enhanced by c-Kit^+^ ASCs, compared to the other culture groups. (e) The c-Kit^+^/c-Kit^−^ ASCs had no potential of tube formation compared with EPCs. ^*∗*^*p* < 0.05; ^*∗∗*^*p* < 0.01; ^*∗∗∗*^*p* < 0.001. (e): 200x magnification.

To test the effects of c-Kit^+^ ASCs on the viability and proliferation of breast cancer cells, we cocultured ASCs with 4T1 cells. With a direct coculture model, we found that the mRNA expression of c-Kit was significantly higher in the coculture group than in the c-Kit^+^ ASCs alone group (*p* < 0.001, Figure 2(a)). Western blot analysis showed no detectable c-Kit protein in the 4T1 cells alone group, but there was a higher c-Kit protein level in the coculture group than in the c-Kit^+^ ASCs alone group (Figure 2(b)). However, we did not observe c-Kit expression in the c-Kit^−^ ASCs alone or coculture group, which suggests that 4T1 cancer cells may increase c-Kit expression in c-Kit^+^ ASCs (Figure 2(b)).

To assess the effect of ASCs on 4T1 cell proliferation, we performed an indirect coculture of ASCs with 4T1 cells and determined the viability of 4T1 cells for 5 days. After cell culture for 3 days, the viability of 4T1 cells was significantly enhanced in the coculture with c-Kit^+^ ASCs, compared to the 4T1 cell culture alone group (*p* < 0.05, Figure 2(c)). The cell proliferation assay using a Quant-iT PicoGreen kit found that the proliferation of 4T1 cells was significantly increased in the coculture with c-Kit^+^ ASCs, compared to the culture of 4T1 cells alone for 4-5 days (*p* < 0.01, Figure 2(d)). These results suggest that c-Kit^+^ ASCs promote the viability and proliferation of breast cancer cells.

### 3.3. Effect of c-Kit^+^ ASCs on Primary 4T1 Tumor Growth In Vivo

**Figure 3:**
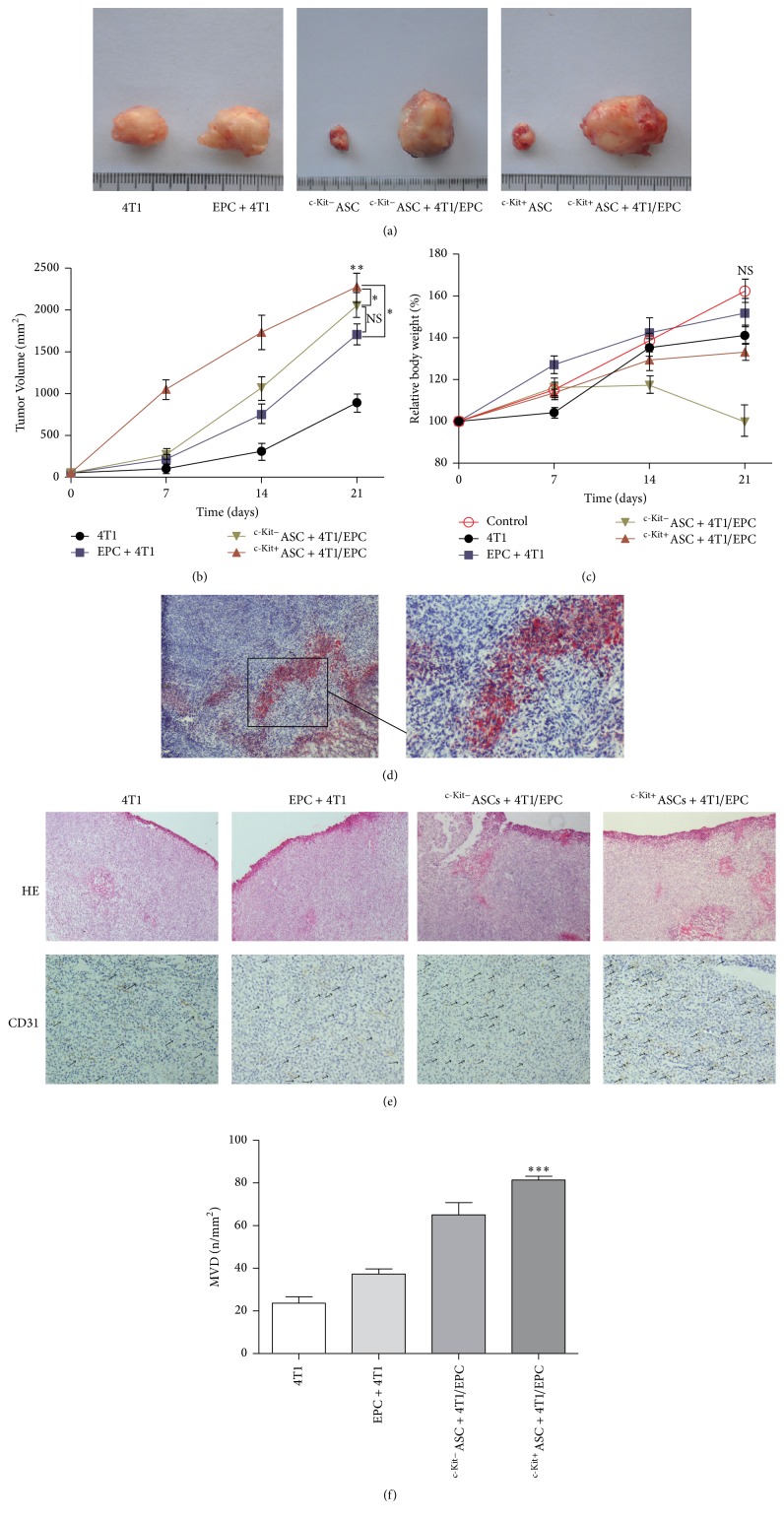
The effect of c-Kit^+^ ASCs on tumorigenesis and angiogenesis of 4T1 breast cancer cells in vivo. c-Kit^+^ ASCs (10^5^) with 4T1 cells (10^5^) and EPCs (10^4^) were suspended in 200 *µ*L of PBS/Matrigel and injected onto mammary fat pads of female nude mice as follows: injection alone or EPCs + 4T1 or ASCs + 4T1/EPCs coinjection. Tumor growth and vessel formation were detected. ((a)-(b)) The primary tumor volume was significantly increased in the c-Kit^+^ ASCs coinjection group, compared with the other injection groups. (c) The weight of the nude mouse increased, and no significant differences between groups, except for a decrease in the c-Kit^−^ ASC coinjection group at 14 days after injection, were observed. (d) Mature adipose formation in the tumor was confirmed by oil red O staining in the ASC coinjection groups. (e) H&E staining and CD31 immunostaining showed broad vessel formation in the tumors, as indicated by the arrows. (f) The microvascular density was higher in the tumors containing c-Kit^+^ ASCs than in the other injection groups. ^*∗*^*p* < 0.05; ^*∗∗*^*p* < 0.01; ^*∗∗∗*^*p* < 0.001. (d) (left) and (e): 40x magnification; (d) (right): 100x magnification. CD31: platelet endothelial cell adhesion molecule-1. MVD: microvascular density.

ASCs affect tissue regeneration and homeostasis, and c-Kit^+^ ASCs have a higher differentiation potential compared to other subpopulations of mesenchymal stem cells in adipose tissue [12, 13]. In addition, tumor growth depends on vessel formation. Nevertheless, our Matrigel assay showed that both c-Kit^+^ and c-Kit^−^ ASCs showed no tube formation ability in comparison to EPCs (Figure 2(e)). To explore whether c-Kit^+^ ASCs promote breast cancer growth in vivo, we performed subcutaneous coinjection of 4T1 cells with ASCs in combination with EPCs into nude mice and observed the effect of c-Kit^+^ ASCs on 4T1 tumor growth. We found that injection of 4T1 cells alone formed a palpable tumor (Figure 3(a)). In the coinjection groups, the tumor growth was markedly stimulated in comparison to the injection of 4T1 cells alone (*p* < 0.01, Figures 3(a) and 3(b)). Furthermore, the tumor volume was significantly increased in the c-Kit^+^ASCs + 4T1/EPCs group, compared with the c-Kit^−^ASCs + 4T1/EPCs group and the EPCs/4T1 group (2275 mm^2^ versus 2052 mm^2^ and 2275 mm^2^ versus 1705 mm^2^, respectively; *p* < 0.05, Figures 3(a) and 3(b)). Although the tumor volume in the c-Kit^−^ASCs + 4T1/EPCs group was larger than that of the EPCs/4T1 group up to 21 days after injection (2052 mm^2^ versus 1705 mm^2^, Figures 3(a) and 3(b)), the difference was not significant (*p* > 0.05, Figure 3(b)). In addition, 14 days after injection, the weights of the nude mice significantly declined by 0.24% of the original weight in the c-Kit^−^ASCs + 4T1/EPCs group (19.14 g versus 19.65 g, Figure 3(c)). However, there was no significant difference of the weight in each group (*p* > 0.05, Figure 3(c)).

### 3.4. c-Kit^+^ ASCs Promote EPC-Mediated Tumor Angiogenesis

Tumor angiogenesis is crucial to tumor growth. However, the role of ASCs in tumor angiogenesis is unclear. To explore the role for c-Kit^+^ ASCs in tumor angiogenesis, tumor grafts were analyzed by H&E staining and mouse-specific anti-CD31 staining. Oil red O staining showed lots of mature adipose cells in the tumor tissues of the ASCs + 4T1/EPCs group (Figure 3(d)); however, H&E and CD-31 immunostaining of tissue sections revealed broad vascularization in the c-Kit^+^ASCs + 4T1/EPCs group, in contrast to the other groups (Figure 3(e)). Quantification analysis revealed that the microvascular density was significantly higher in the coinjection groups containing c-Kit^+^ ASCs than in the other injection groups (81.3 ± 3.1 vessels/mm^2^ versus 65.0 ± 10.0 vessels/mm^2^ in the c-Kit^−^ASCs + 4T1/EPCs group, *p* < 0.05; 37.3 ± 4.2 vessels/mm^2^ in the EPCs/4T1 group, *p* < 0.001; 23.7 ± 5.1 vessels/mm^2^ in the 4T1 group, *p* < 0.001, Figure 3(f)), suggesting that c-Kit^+^ ASCs may enhance EPC-mediated tumor angiogenesis of breast cancer.

### 3.5. c-Kit^+^ ASCs Stimulate the Release of Interleukin-3 (IL-3), Stromal Cell-Derived Factor-1 (SDF-1), and VEGF-A

**Figure 4:**
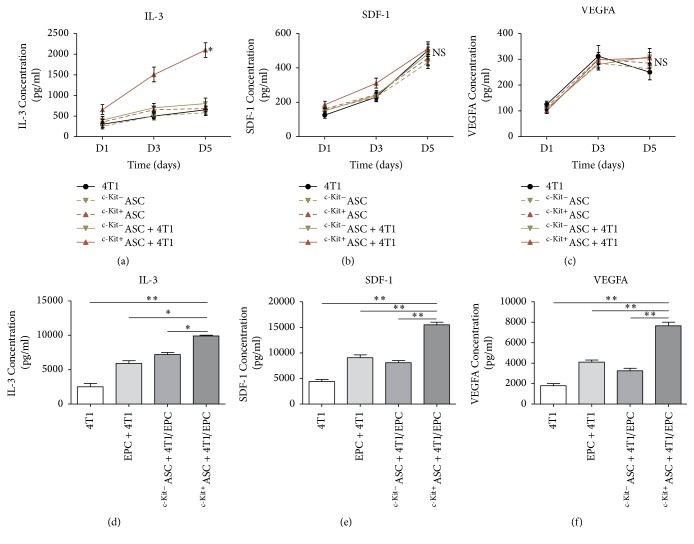
The release of cytokines and chemokines in c-Kit^+^ ASCs. Cell culture supernatant and tumor tissue supernatant were collected and assayed with ELISA. ((a)–(c)) The release of IL-3 was significantly higher in the c-Kit^+^ASCs/4T1 direct coculture group in comparison with single culture; in contrast, there was a significant difference between the SDF-1 and VEGF-A levels among the groups. ((d)-(e)) The release of IL-3 as well as SDF-1 and VEGF-A was significantly increased in the coinjection group of EPCs/4T1 with c-Kit^+^ ASCs, compared with the other groups. ^*∗*^*p* < 0.05; ^*∗∗*^*p* < 0.01.

ASCs produce several cytokines and chemokines that affect other surrounding cells, especially EPCs, thereby increasing tumor angiogenesis. To explore the molecular mechanisms by which c-Kit^+^ ASCs enhance EPC-mediated tumor angiogenesis of breast cancer, we analyzed the relevant cytokines, chemokines, and angiogenic growth factors. In direct coculture models, ELISA analyses revealed that the release of IL-3 significantly increased in the c-Kit^+^ASCs/4T1 group in comparison to the c-Kit^−^ASCs/4T1 group (1416.7 ± 728.6 pg/mL versus 633.3 ± 208.2 pg/mL, *p* < 0.05, Figure 4(a)) and the single culture groups (483.3 ± 175.6 pg/mL in the 4T1 group, *p* < 0.05; 443.3 ± 172.1 pg/mL in the c-Kit^−^ASCs group, *p* < 0.05; 560.0 ± 182.5 pg/mL in the c-Kit^+^ASCs group, *p* < 0.05; Figure 4(a)). However, there was no significant difference in the SDF-1 (*p* > 0.05, Figure 4(b)) and VEGF-A (*p* > 0.05, Figure 4(c)) levels among the groups.

To extend our observation in vitro to mouse models in vivo, the levels of IL-3, SDF-1, and VEGF-A were assessed in tumor tissues. Interestingly, we found that IL-3 was significantly increased in the c-Kit^+^ASCs + 4T1/EPCs group, compared with the c-Kit^−^ASCs + 4T1/EPCs group (9900.0 ± 141.4 pg/mL versus 7250.0 ± 353.6 pg/mL, *p* < 0.05, Figure 4(d)) and the other groups (5900 ± 565.7 pg/mL in the EPCs/4T1 group, *p* < 0.05; 2500 ± 707.1 pg/mL in the 4T1 group, *p* < 0.01, Figure 4(d)). Moreover, the release of SDF-1 significantly increased in the c-Kit^+^ASCs + 4T1/EPCs group in comparison with the c-Kit^−^ASCs + 4T1/EPCs group (15500.0 ± 707.1 pg/mL versus 8150.0 ± 495.0 pg/mL, *p* < 0.01, Figure 4(e)) and the other groups (9050.0 ± 777.8 pg/mL in EPCs/4T1 group, *p* < 0.01; 4400.0 ± 565.7 pg/mL in the 4T1 group, *p* < 0.01, Figure 4(e)). In addition, the level of VEGF-A was significantly higher in the c-Kit^+^ASCs + 4T1/EPCs group than in the c-Kit^−^ASCs + 4T1/EPCs group (7650.0 ± 495.0 pg/mL versus 3250.0 ± 353.6 pg/mL, *p* < 0.01, Figure 4(f)) and the other groups (4150.0 ± 212.1 pg/mL in EPCs/4T1 group, *p* < 0.01; 1800.0 ± 282.8 pg/mL in the 4T1 group, *p* < 0.01, Figure 4(f)). However, there were no significant differences between the c-Kit^−^ASCs + 4T1/EPCs group and the EPCs/4T1 group in terms of the IL-3, SDF-1, and VEGF-A levels (*p* > 0.05, Figures 4(d)–4(f)).

## 4. Discussion

ASCs have been proposed to promote the viability of fat grafts and the efficacy of the procedure after breast cancer surgery [23, 24]. However, emerging evidence indicates that ASCs may contribute to the growth and metastasis of breast cancer [25, 26]. Conversely, other studies have demonstrated an inhibitory role of ASCs in breast cancer [6, 7]. The contradiction warrants the selection of immunophenotypic markers of ASCs in clinical application. Our results demonstrated that c-Kit^+^ cells with characteristics of adipogenesis could be isolated from ASCs and that the c-Kit^+^ subpopulation was very rare, which is consistent with published observations that most stromal vascular fraction cells are c-Kit^−^ [27–29].

There are distinct expression levels of c-Kit in stem cells/progenitor cells, melanocytes, and mast cells [30]. The c-Kit signaling network supports the proliferation, differentiation, and survival of c-Kit-expressing cells [31, 32]. In BRCA1 mutation-associated breast cancers, c-Kit is required for the growth and survival of the tumor cells, and c-Kit activity may be downregulated to allow normal differentiation in adult tissue [33]. At present, to the best of our knowledge, no studies have shown the effect of c-Kit^+^ ASCs on breast cancer progression compared with the other subpopulations of ASCs. In the present study, we showed that the expression of c-Kit increased in the c-Kit^+^ASCs/4T1 coculture group compared with single cultures and that the proliferation of breast cancer cells was enhanced by c-Kit^+^ ASCs after coculture. Moreover, the release of membrane-bound KitL, as an adhesion/survival-promoting molecule for stem cells, depends on IL-3 [34]. IL-3 acts as a nonspecific proinflammatory cytokine and drives cell proliferation by the JAK/STAT and PI3K/AKT pathways [35–37]. Our results demonstrated that c-Kit^+^ ASCs stimulated IL-3 release both in vitro and in vivo. Taken together, our results suggest that c-Kit may stimulate the proliferation of 4T1 breast cancer cells by promoting IL-3 release.

Tumor angiogenesis is crucial to tumor growth [38–40]. However, we found that the ASCs had no tube formation ability in vitro, and no palpable tumor masses formed in the ASCs injection alone group in mice. A previous study has shown the perivascular properties of ASCs in stabilizing these neovessels and the effect on EPC/endothelial cells by paracrine signaling in vivo [41]; accordingly, we implanted ASCs together with the EPCs that were isolated and cultured in the same conditions in order to eliminate their effect. Interestingly, we observed that c-Kit^+^ ASCs significantly promoted tumor growth in combination with EPCs, compared to the c-Kit^−^ ASCs coinjection group and the other groups, especially at 1 week after injection. Tumor angiogenesis is sustained by complicated cytokine networks among tumor cells and other cells in the tumor microenvironment [42–45]. We found that c-Kit^+^ ASCs had an intrinsic proangiogenic capability and stimulated IL-3 release, which could promote the proliferation of 4T1 breast cancer cells or the growth of mature adipose cells. However, the tumor microenvironment in solid tumors is hypoxic, and c-Kit has been reported to participate in the formation of the tumor vasculature via promoting HIF-1*α*-mediated VEGF expression [46, 47]. Boesiger et al. have shown that, in response to c-Kit/KitL, mouse or human mast cells rapidly release VEGF by degranulation and then sustain the release by secreting newly synthesized proteins [48]. Meanwhile, an increase of angiogenic factors, such as HIF-1*α*, SDF-1, and VEGF, in turn causes activation of matrix metalloproteinase-9 and thus initiates recruitment and mobilization of BM-EPCs in the peripheral circulation [49]. Our results indicate that active c-Kit^+^ ASCs may recruit myeloid cells by SDF-1 for angiogenesis, thereby stimulating EPCs to form vessels followed by the release of VEGF-A, which are crucial factors for the proliferation of breast cancer cells in the tumor microenvironment [50, 51].

Accumulating evidence suggests that ASCs may favor tumor progression by autocrine and paracrine signaling in different transplanted and metastatic tumor models. However, the interaction mechanism between ASCs and breast cancer in the tumor microenvironment remains to be determined. One limitation of our current study is the lack of continuous observation and cell tracking to discriminate different cell types and illuminate the specific role of cells in tumor angiogenesis in every group. Moreover, human tissue-derived c-Kit^+^ ASCs are needed to prove the function of c-Kit^+^ ASCs in breast cancer progression. In addition, multicenter clinical outcomes based on an adequate follow-up of breast cancer patients with an autologous fat graft are needed to compare c-Kit^+^ ASCs with other subpopulations of ASCs.

In conclusion, c-Kit^+^ ASCs can promote the tumor angiogenesis and growth of breast cancer by recruiting EPCs via a synergistic effect of c-Kit and IL-3. Our findings suggest that identification of different phenotypes of ASCs may be required in reconstructive efforts with stem cell-enhanced fat grafting. Furthermore, c-Kit^+^ ASCs should be avoided for use in breast reconstruction.
